# Desquamative Gingivitis in the Context of Autoimmune Bullous Dermatoses and Lichen Planus—Challenges in the Diagnosis and Treatment

**DOI:** 10.3390/diagnostics12071754

**Published:** 2022-07-20

**Authors:** Ana Maria Sciuca, Mihaela Paula Toader, Carmen Gabriela Stelea, George Alexandru Maftei, Oana Elena Ciurcanu, Ovidiu Mihail Stefanescu, Bianca-Andreea Onofrei, Cristina Popa

**Affiliations:** 1 Discipline of Oral Medicine, Oral Dermatology, Grigore T. Popa University of Medicine and Pharmacy, 16 Universitatii Str., 700115 Iasi, Romania; afilioreanu@yahoo.com (A.M.S.); george-alexandru.maftei@umfiasi.ro (G.A.M.); andreea.turcanu@yahoo.com (B.-A.O.); cristina.popa@umfiasi.ro (C.P.); 2 Discipline of Oral Surgery, Grigore T. Popa University of Medicine and Pharmacy, 16 Universitatii Str., 700115 Iasi, Romania; oana.ciurcanu@umfiasi.ro (O.E.C.); ovidiu.stefanescu@umfiasi.ro (O.M.S.)

**Keywords:** desquamative gingivitis, autoimmune bullous diseases, pemphigus vulgaris, bullous pemphigoid, cicatricial pemphigoid, lichen planus

## Abstract

Desquamative gingivitis (DG) is a clinical term that describes erythema, desquamation and erosions of the gingiva, of various etiologies. Although the clinical aspect is not specific for a certain disease, an accurate diagnosis of the underlying disorder is necessary because the disease course, prognosis and treatment vary according to the cause. DG may inflict significant oral discomfort, which is why patients typically present to the dentist for a first consultation, rendering it important for these specialists to be informed about this condition. Our paper aims to review the ethiopatogenesis and diagnostic approach of DG, focusing on the most common underlying disorders (autoimmune bullous dermatoses and lichen planus) and on the management of these patients. Potential etiological agents leading to an inflammatory immune response in the oral mucosa and DG appearance include genetic predisposition, metabolic, neuropsychiatric, infectious factors, medication, dental materials, graft-versus-host reaction and autoimmunity. A thorough anamnesis, a careful clinical examination, paraclinical explorations including histopathological exam and direct immunofluorescence are necessary to formulate an appropriate diagnosis. Proper and prompt management of these patients lead to a better prognosis and improved quality of life, and must include management in the dental office with sanitizing the oral cavity, instructing the patient for rigorous oral hygiene, periodic follow-up for bacterial plaque detection and removal, as well as topical and systemic therapy depending on the underlying disorder, based on treatment algorithms. A multidisciplinary approach for the diagnosis and follow-up of DG in the context of pemphigus vulgaris, bullous pemphigoid, cicatricial pemhigoid or lichen planus is necessary, including consultations with dermatologists, oral medicine specialists and dentists.

## 1. Introduction

The word “desquamative” originates from the latin “desquamare”, which means to scrape fish scales. Ad literam, desquamation involves the loss of epithelium, exfoliation of the skin or mucous membranes [1].

Desquamative gingivitis (DG) is a descriptive clinical term characterized by intense erythema, desquamation, vesicles or bullae leading to erosions or ulcers, involving the free or attached gingival mucosa. It was first mentioned by Tomes in 1894. In 1932, Prinz established it as a descriptive term for erythema, desquamation, erosions and blisters on the marginal and attached gingiva. Following Mc Carthy’s hypothesis from 1960, Glickman and Smulowin demonstrated in 1964 that DG is a clinical manifestation of several diseases [1,2]. The most common causes are dermatological conditions such as lichen planus (LP) in 70–75% of cases, cicatricial pemphigoid (CP) in 9–14% of cases and pemphigus vulgaris (PV) in 4–13% of cases [3]. Hypersensitivity reactions triggered by contact allergens in mouthwashes, toothpastes, dental materials or medication may also lead to DG [2,4,5,6]. Other causes are represented by paraneoplastic pemphigus, bullous pemphigoid, linear IgA disease, lichen planus pemphigoides, lupus erythematosus, mixed connective tissue disease, epidermolysis bullosa, epidermolysis bullosa acquista, graft-versus-host disease, erythema multiforme, plasmacytosis, plasma cell gingivitis, orofacial granulomatosis, Kindler syndrome, foreign body granulomas, chronic ulcerative stomatitis, endocrine disorders (estrogen deficiencies, testosterone imbalance, hypothyroidism) and chronic bacterial infections [7,8,9,10,11,12,13,14,15]. Systemic disorders such as Crohn’s disease, sarcoidosis or psoriasis and even COVID-19 may lead to desquamative gingivitis-like manifestations [4,16,17,18,19,20].

Our narrative review focuses on the most common underlying diseases manifesting with DG, namely autoimmune bullous dermatoses and lichen planus. Autoimmune bullous diseases are a group of morbid conditions with different etiology, pathogenesis and prognosis that affect the skin and/or mucous membranes. The gingival fibromucosa is often the site of onset of these diseases. Subsequently, the clinical manifestations may spread to the skin or to other mucous membranes (conjunctival, nasal, pharyngeal, laryngeal, esophageal or genital) [15,19]. Lichen planus is a chronic inflammatory mucodermatosis with an autoimmune mechanism, often with oral manifestations, which can take on, in some forms, the character of DG [2].

As DG is often the first, if not the only sign of disease, leading to significant oral discomfort, patients commonly go to the dentist for a first consultation. It is, thus, important for these specialists to know how to diagnose the condition, as early adequate treatment is essential in DG associated with autoimmune diseases, and a multidisciplinary approach is often required for the appropriate management of these patients.

The clinical appearance of DG is not specific for a certain disease. However, there are several associated clinical findings that may point to an accurate diagnosis, which is essential, because the disease course, treatment and prognosis are different depending on the cause. The systemic involvement and the degree of oral mucosal damage are important factors that contribute to the general morbidity and mortality associated with autoimmune disorders, as well as to the overall quality of life in these patients [1,21].

## 2. Etiopathogenesis

DG is predominantly observed in women, with a female to male ratio of 4:1. It occurs more commonly in middle-aged and older adults, and only in rare cases in children [8,22].

A multitude of factors have been proposed as potential etiological agents involved in the development of the inflammatory immune response in the oral mucosa leading to a clinical appearance of DG (Table 1).

### 2.1. Genetic Predisposition

DG lesions in lichen planus, for example, have a family incidence of 1–15% and are associated with HLA-B7. The onset is early, the lesions are recurrent and the response to treatment is lower [3].

### 2.2. Metabolic Factors

The association with hypertension, diabetes and dyslipidemia is mentioned [3,8].

### 2.3. Neuropsychiatric Factors

It has been noted that gingival changes can be triggered by an emotional shock in patients with a particular psychological profile. Anxious, depressed individuals, with episodes of insomnia, or other associated neurological disorders, are frequently affected [3,8]. 

### 2.4. Medication

Several drugs, such as NSAIDs, antihypertensives, methyldopa, allopurinol, ketoconazole etc. cause the formation of scaly lesions in the mucous membranes [3,6,8].

### 2.5. Infectious Factors

Several studies show an increased prevalence of liver disease in patients with chronic DG. Up to 20% of them are associated with hepatitis C virus (HCV) or hepatitis B virus (HBV), with viruses replicating in the oral mucosa. In patients receiving interferon and ribavirin therapy, the oral lesions either remit or are exacerbated. An association with human papilloma virus (HPV) has also been reported, as specific T lymphocyte clones were observed in these patients. Helicobacter pylori also induces the formation of proinflammatory interleukins and cytokines, involved in the pathogenesis of oral lesions. Tuberculosis, histoplasmosis and chronic candidiasis are also cited as a potential cause for DG [1,2,3,8].

The accumulation of microorganisms around the teeth in close proximity to the gingiva, leading to the formation of dental plaque, induces the release of proinflammatory cytokines, the recruitment of inflammatory cells, and periodontal inflammation. Whether periodontal inflammation is a factor in the onset of DG or merely contributes to its progression is still a subject of debate [8,23,24,25,26].

### 2.6. The Graft-versus-Host Reaction 

This is a dreaded complication of transplantation, and oral damage is present in 33–75% of patients who develop it [27].

### 2.7. Certain Dental Materials 

Certain dental materials used in dentistry, such as amalgam, gaudent, cobalt and nickel can cause an allergic reaction. Because the allergens involved are mixed with saliva, the mucosal reaction can extend away from the contact area [8,28].

### 2.8. Autoimmunity

Up to 27% of cases of DG occur in association with CP or PV. As triggering factors for the onset of these diseases, smoking, alcohol consumption, immunosuppression, drug abuse, neoplasms, infectious factors and local trauma have been mentioned [3].

Autoimmune bullous disorders are characterized by the presence of autoantibodies against antigens in the epithelium or the basement membrane. These antigens are represented by proteins of intercellular and membrane-cell adhesion structures. The different topography of numerous antigens in the epidermis or basement membrane explains the presence of intra- or subepithelial bullous lesions and they are specific to certain morbid conditions with different prognosis and treatment [2,19,29]. Table 2 shows the target antigens specific for the most common autoimmune bullous diseases with oral involvement.

Frequently, DG also appears as a consequence of an association of diseases, for example oral LP associated with other autoimmune conditions, such as lupus erythematosus, scleroderma, pemphigoid, dermatomyositis etc. [2,3,8].

## 3. Diagnostic Approach

### 3.1. Clinical Findings

DG presents with an association between two or more of the following: erythema, hyperkeratosis, desquamation, atrophy, blisters, erosions and ulcers.

Nisengard and Levine cited the following clinical criteria in diagnosing DG:

(1) non-plaque gingival erythema;
(2) gingival desquamation;
(3) the presence of other intraoral and sometimes extraoral lesions;
(4) pain, especially when eating spicy foods.

The symptoms depend on the type and extent of the lesions. Mild discomfort or pain is usually reported with keratosis and atrophy. Moderate pain occurs when erosions are present, or blisters form. Patients avoid spicy foods, sour drinks and alcohol, which exacerbate the pain. Severe pain is associated with ulceration and large areas with missing epithelium, leading to the patients’ inability to feed. Dehydration is a severe complication as a result of pain with mastication and reduced oral food and fluid intake, requiring hospitalization [22,30].

### 3.2. Patients’ History

Age of the patient is important, the majority of autoimmune diseases manifesting in middle-aged or older adults, with only a minority of cases in children. Family history must be inquired into as genetic predisposition is established in most autoimmune diseases. Potential triggering factors such as alcohol consumption, smoking, local trauma, dental restorative procedures, chronic infections, endocrine disturbances, metabolic disorders, transplants and medication must be taken into account. Time of onset, evolution (single or recurrent episodes), duration of disease, as well as associated cutaneous lesions must be searched for [3,30,31].

### 3.3. Paraclinical Explorations

A correct diagnosis is difficult to make in the absence of complementary examinations. In most cases, a biopsy of the affected tissue is performed, the pathological examination and direct immunofluorescence being required for a definite diagnosis [1]. In case of suspected autoimmune bullous disorders the protocol for paraclinical examinations includes Tzanck smear, pathological examination, electron microscopy, direct and indirect immunofluorescence [7,12,26,32,33]. 

The biopsy specimen must include an area of intact epithelium, as well as perilesional tissue. This may require two separate biopsies, one from the lesion and the perilesional area and one from normal appearing mucosa. Direct immunofluorescence (DIF) should be performed on biopsy specimens from normal-appearing tissue rather than perilesional sites in suspected autoimmune diseases such as CP, BP and PV, as immune deposits in these morbid conditions are present in all oral tissue and the pattern of distribution of immune deposits is more clearly seen in intact epithelium [32,33,34,35,36,37,38].

Recently, Optical Coherence Tomography (OCT) has been proposed as a preliminary non-invasive diagnostic tool for the most frequent immune-mediated diseases that can cause DG [39]. This diagnostic tool has been observed to display particular patterns in the case of DG-mediated pemphigus vulgaris (such as diminished epithelial thickness, intraepithelial unilocular blister and acantholytic cells present in the blister) and in the case of mucous membrane pemphigoid (for example existence of inflammatory infiltrate and multilocular subepithelial blister). Furthermore, OCT offers the possibility to deposit and examine images at any time, facilitating treatment surveillance and the identification of disease recurrence prior to clinical manifestations [39,40]. Even though research is ongoing, OCT could pose an intriguing non-invasive alternative for clinical preliminary evaluation and surveillance of such intricate pathological conditions.

## 4. Associated Autoimmune Bullous Dermatoses

### 4.1. Pemphigus Vulgaris (PV)

PV is an autoimmune bullous disease that affects the skin and oral and genital mucosa. It is characterized by the production of IgG and IgA autoantibodies against desmosomal proteins—desmogleins 3 and 1 (DSG1, DSG3), with acantolysis and blister formation, leaving secondary erosions [26].

PV is a rare disease, with a prevalence of 94.8 per million inhabitants in Germany in 2014. The annual incidence, estimated in European countries, is in the range of 0.7–8 cases per million inhabitants and it is the highest in patients living in southeast European countries. On average, patients develop PV in the fifth decade of life, with a slight predominance among women [41].

The pathogenesis of PV involves IgG autoantibodies directed against DSG1 and DSG3 proteins. Genetics appears to contribute to the pathogenesis of the disease, with HLA class II genes being described, as well as an association with non-HLA genes, the most commonly reported being HLA-DQB1 * 0503 and DRB1 * 0402 alleles, expressed in antigen presenting cells [42,43]. 

Most patients with PV in which DSG3 autoantibodies predominate develop a phenotype with predominant mucosal involvement, while patients with autoantibodies to both DSG1 and DSG3 have mucocutaneous lesions. DSG3 is expressed only in the basal layers of the skin, while DSG1 is expressed in the entire epithelium, compensating for the loss of DSG3 function. In the mucosa, DSG3 is expressed throughout the epithelium, while DSG1 is predominantly expressed in the superficial layers, and therefore the loss of DSG3 cannot be adequately compensated by DSG1. Exclusive reactivity to DSG3 causes only mucosal lesions [44,45].

The oral mucosa is the first to be affected in 50–80% of patients. Oral lesions often precede skin lesions or are the only manifestations of the disease [26]. Mucocutaneous PV is more common than mucous membranes PV. Involvement of the oral mucosa in pemphigus vegetans, a clinical variant of PV, is reported in 60–80% of cases [42,46].

**Figure 1:**
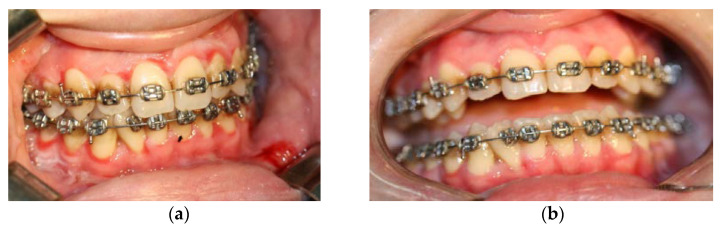
DG in a patient with PV. The patient was not allergic to nickel, and did not have a history of other allergies. (**a**) Intense erythema, desquamation and erosions of the attached gingiva—before treatment. (**b**) Remission of erythema and healed erosions after systemic corticosteroid treatment.

Skin lesions usually consist of superficial, flabby blisters, which break easily, leaving erosions and crusts, and are usually distributed on the face, scalp and upper chest. DG is seen in about a quarter of patients with PV (Figure 1). The oral mucosa is often eroded, with rapid rupture of superficial blisters so that intact blisters are rarely seen and healing is done without scarring. The palatine mucosa, jugal mucosa, labial mucosa and tongue are the most commonly affected. Other mucous membranes may be involved, including the less common pharyngeal and nasal mucosa, genital, ocular, laryngeal and esophageal mucosa [46].

The diagnosis is based on the clinical appearance of the lesions corroborated with paraclinical examinations. Tzanck cytodiagnosis reveals acantolytic cells, i.e., segregated and dysmorphic keratinocytes, with shape and volume anomalies, monstrous nucleus and diminished cytoplasm. Histopathological examination reveals the presence of slit spaces above the epithelial basal layer. Acantolytic cells are present inside these slits. In some cases, blistering is preceded by eosinophilic spongiosis. Electron microscopy shows widening of the intercellular spaces, rupture of desmosomal junctions and retraction in the perinuclear area of keratin tonofilaments [45].

Direct immunofluorescence, on a loose biopsy fragment of skin or mucosa, identifies IgG deposits (IgG1 and IgG4) on the surface of keratinocytes, at and near the lesions. It is the most sensitive method of diagnosing oral pemphigus.

Indirect immunofluorescence allows the detection of circulating antibodies (IgG), in the serum of 80–90% of patients and uses as a substrate a squamous stratified epithelium (monkey esophageal epithelium) [7,26].

Delaying diagnosis affects the prognosis, increases the risk of infection and the length of hospitalization and treatment, and decreases the quality of life of patients [26].

The observed mortality rates are 2–3.3 times higher compared to the general population, with survival rates of 95% at 1 year, 93% at 5 years and 90% at 10 years. Infection is the most common cause of death, especially pneumonia and sepsis [46].

Systemic treatment of PV aims to improve symptoms, based on the use of corticosteroids and immunosuppressants. Systemic corticosteroids are essential for the management of pemphigus vulgaris and may be combined with other immunosuppressants (azatrioprine, mycophenolate mofetil, rituximab). Oral lesions are difficult to manage and can be treated with topical corticosteroids, including betamethasone and triamcinolone acetonide [47].

**Figure 2:**
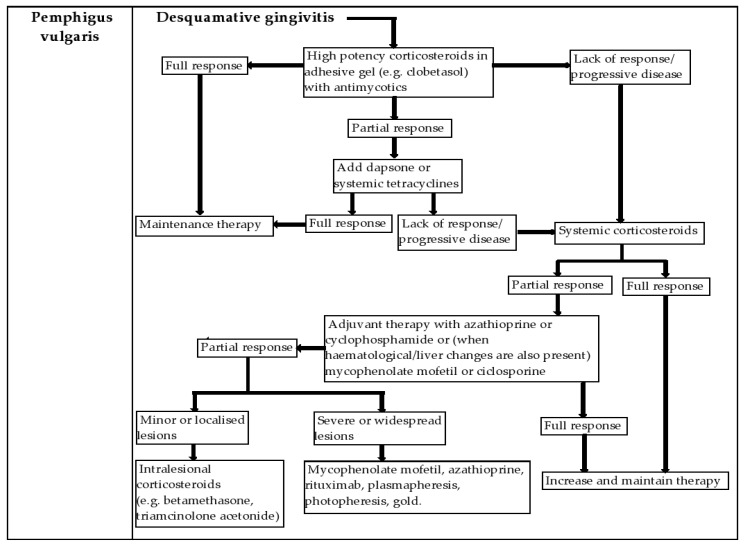
Treatment algorithm for desquamative gingivitis due to pemphigus vulgaris.

Maintenance of a rigorous oral hygiene is hampered by the presence of painful lesions, which causes secondary aggravation by bacterial superinfection of pre-existing periodontal disease [48,49]. Figure 2 is a summary of the treatment algorithm for desquamative gingivitis due to pemphigus vulgaris.

### 4.2. Bullous Pemphigoid (BP)

BP is a chronic, subepidermal bullous autoimmune disorder [7], which mainly affects people between the ages of 60 and 80, characterized by autoantibodies against two antigens from the hemidesmosomes [12,41,50].

BP has an incidence in the general population between 2.4 and 21.7 per million per year [9], with a slight predominance of females (1.7:1) [7].

The pathogenesis is characterized by an autoimmune process in which autoantibodies (IgG/IgE type) target two different proteins: the alpha-1 (XVII) collagen chain (formerly known as BP180/BPAG2) and dystonine (formerly known as BP230/BPAG1) in the basement membrane. The degradation of the alpha-1 collagen chain (XVII) is considered essential in the process of blister formation, followed by the activation of the complement and subsequently of the inflammatory cascade. The NC16 domain appears to be the target epitope in most patients with BP. Histologically, the damaged and adjacent skin shows the detachment of the basal keratinocytes of the epidermis from the dermal tissue—in the lamina lucida. Direct immunofluorescence exposes immune deposits (IgG or C3) with specific (linear) deposition in the basement membrane. In direct jump-split immunofluorescence, IgG/C3 immune deposits are observed on the roof of the blister [51,52].

Exposure to various drugs such as dipeptyl peptidase-4 inhibitors (DPP-4) and protein-1 inhibitors of programmed cell death, as well as coexistence with a number of other immunological diseases such as diabetes, psoriasis, lichen planus and pernicious anemia, may cause the appearance of BP [51]. BP has been frequently associated with hematological or solid neoplasms but the relationship between these entities is controversial [7,11].

Clinically, BP is characterized by the presence of large, tense blisters that may begin in the form of erythematous macules, urticarial papules, or plaques [3] electively located on the trunk and limbs, but there may be atypical lesions involving the mucous membranes [53].

Involvement of the oral mucosa is rare, but 10–20% of patients have lesions at this level usually in the form of erosions (very rarely in the form of a blister). The blisters rupture very quickly, forming erosions, especially affecting the jugal mucosa, palate, gums, tongue and lower lip. In general, BP is characterized by slow-growing, smaller blisters that are not as painful as lesions in PV. Gums can be affected in 16% of cases causing DG. The condition is chronic, with recurrent episodes [7,54].

Chuah et al. consider that the involvement of the oral mucosa in newly diagnosed patients is associated with a more difficult response to conventional treatments, recommending caution in the choice of treatment (the intention to use adjuvant therapies) [55].

The diagnosis of this disease is based on the clinically suggestive appearance of the lesions (large, tense blisters that do not extend peripherally and have a spontaneous tendency to heal), histopathological examination (subepidermal blister, lack of acantholysis), direct immunofluorescence (linear IgG/C deposits in the basement membranel) and serological investigations. Serum autoantibody levels can be detected using enzyme immunoassays (ELISA) [53,56]. There are insufficient studies in the literature to correlate the clinical severity of the disease with the level of antibodies in the serum with the presence of proteins in the basement membrane.

**Figure 3:**
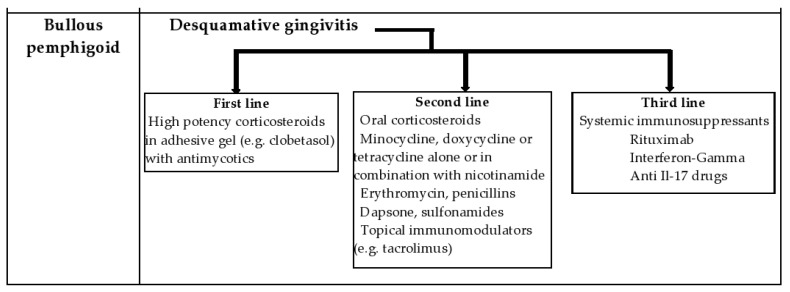
Treatment algorithm for desquamative gingivitis due to bullous pemphigoid.

The prognosis is influenced by advanced age, the presence of comorbidities, the severity of the disease and the type of treatment [57]. Treatment choice depends on the activity and severity of the disease, but includes glucocorticoids and other immunosuppressants, with intravenous immunoglobulin treatment being considered when the condition is not controlled [58]. Patients with limited forms of disease respond well to topical corticosteroid therapy, particularly when the oral mucosa is the only one affected. Those with extensive forms of the disease require systemic corticosteroids and immunosuppressive agents. New therapeutic approaches for treatment-refractory BP include Rituximab, Interferon-Gamma, and anti Il-17 drugs (Secukinumab, Ixekizumab) [56,58,59]. Figure 3 is a summary of the treatment algorithm for desquamative gingivitis due to bullous pemphigoid.

### 4.3. Cicatricial Pemphigoid (CP)

CP is an autoimmune bullous, chronic condition with subepithelial blisters, which occurs more frequently in women and elderly patients, without a geographical or racial preference [12,60].

The pathogenesis of CP is complex, being a heterogeneous condition, with the involvement of multiple antigens. Antibodies produced in CP were detected both in vivo and in vitro. In general, antibodies are of the IgG and IgA type, directed against structures in the basement membrane area, which indicates that the appearance of the CP is mediated by a humoral immune response. Loss of tolerance to basal membrane protein morphology leads to the formation of autoantibodies. Through immunoprecipitation and immunoblotting techniques, various structures in the basement membrane have been identified as targets of autoantibodies. Bullous pemphigoid antigen 2 (BPAg2) (a 180 kDa protein, BP180) is the most commonly targeted autoantigen in CP. A reaction of autoantibodies against antigens causes the epithelium to detach from the inside of the basement membrane [12,61,62].

CP can affect various mucous membranes, rarely involving the skin. It is a chronic disease that most commonly affects the oral mucosa, followed by the conjunctiva, nasal mucosa, genital mucosa and gastroesophageal mucosa. The lesions always heal with scars [61].

The disease differs in severity, ranging from localized to diffuse forms. Initially, the condition may be localized, and later there may be extensive involvement. Because the lesions heal with scars, it has a significant morbidity, especially in patients with ocular damage [12].

Oral lesions occur frequently in the gums and palate, followed by the labial mucosa and tongue. These lesions may appear as erythema, pseudo-membrane, erosions and less often intact blisters. Gingival damage is manifested as DG, which is not specific for CP, having a similar appearance in oral lichen planus and pemphigus vulgaris [12].

The diagnosis requires histopathological examination of the tissue samples, which must be taken with the utmost care, as the epithelium can easily detach from the underlying connective tissue. H&E stained sections typically show subepithelial cleavage accompanied by a chronic inflammatory infiltrate. Another piece can be harvested from the perilesional tissue, swabbed in hypertonic saline and examined in direct immunofluorescence. It typically shows linear deposition of IgG, C3 and sometimes IgA along the basement membrane [12,62].

Other paraclinical examinations may be required to determine the titer of serum antibodies, such as indirect immunofluorescence test and the ELISA test which has a higher accuracy and specificity. These tests are useful in diagnosing, but especially in monitoring the evolution of the disease and the response to therapy because the high values of serum antibodies titer are directly proportional to the acute/exacerbation stage of the disease.

CP therapy includes early treatment of lesions and aims to prevent complications especially when there is eye damage. The appearance of scars can be prevented by early therapeutic interventions. The treatment is individualized to each patient, depending on the severity of the disease, taking into account the age, general condition of the patient, pre-existing conditions and contraindications to the use of systemic drugs. The multidisciplinary approach by a team of experts in oral medicine, dermatologists, ophthalmologists and gastroenterologists certainly leads to improved results [12,62].

High potency topical glucocorticoids remain the mainstay of therapy. Fluocinonid, clobetasole propionate and betamethasone dipropionate are the most indicated drugs. Gingival desquamation as the only manifestation can be well managed by using only topical glucocorticoids. However, their long-term use can lead to side effects such as candidiasis, so concomitant prescription of topical antifungals (a combination of clobetasol and miconazole) is required [12,62].

**Figure 4:**
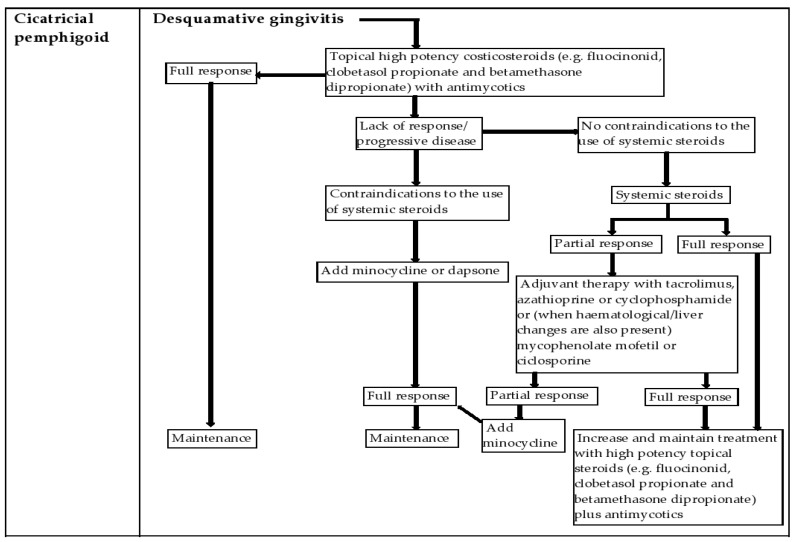
Treatment algorithm for desquamative gingivitis due to cicatricial pemphigoid.

Intralesional and systemic glucocorticoids are used to treat more severe forms, or as an adjunct to topical steroids. Patients who do not respond to steroids may be given strong calcineurin inhibitors—immunosuppressants such as tacrolimus, with a good safety and efficacy profile [12,47] (Figure 4).

Palliative care may also be needed to alleviate the discomfort during mastication. Local anesthetics, analgesics, antihistamines, gels or mouthwashes may be used to improve the patient’s ability to eat, swallow and perform oral hygiene [63].

Differential diagnosis of DG in autoimmune bullous disorders: gingivitis caused by wearing dentures, gingivitis from various viral infections (e.g., herpetic gingivitis) and gingivitis from hematological conditions (e.g., gingivitis from leukemias) [1]. In Table 3 we present the differential diagnoses that are required, in the case of patients with BP, CP and PV.

Although both BP and CP have skin and mucosal lesions, they differ as follows: in BP, skin lesions predominate in the clinical picture, while CP is characterized by predominant mucosal involvement. BP blisters often heal without scarring, being a self-limiting condition, unlike CP, which has a chronic course and the healing of the lesions is sometimes accompanied by a loss of substance. The diagnosis of CP with bullous skin lesions or the diagnosis of BP with extensive oral lesions remains a challenge, especially in the presence of a patient with anti-BP180 autoantibodies present [53].

## 5. Lichen Planus (LP)

LP is a relatively common chronic inflammatory disease, with a T cell mediated mechanism, of unknown etiology that affects the mucous membranes, skin and nails. Its prevalence varies in epidemiological studies between 0.5–2.2% of the population, with a maximum incidence in the range of 30–60 years and with a predominance in females of 2:1. Mucosal LP shows a chronic evolution with acute exacerbations. Spontaneous remission of oral lichen planus (OLP) is less common; on the contrary, oral lesions may worsen over time. It is important to identify cases that may be induced by drugs or that may be associated with an allergic or irritant contact reaction (lichenoid reaction) in order to remove the causative agent. There is a very low risk of malignancy (approximately 1:200 patients/year) associated with OLP, so patients should be monitored long-term by their dentist [64,65,66].

In the case of oral lesions, the pathogenic mechanisms are not yet fully elucidated. However, current data suggest that it is a cell-mediated autoimmune disease. The hypothesis is also supported by the association with other pathologies that are associated with an immune background. There is a T-cell-mediated immune response in which CD8 + lymphocytes trigger apoptosis of the basal cells of the oral epithelium. They recognize a specific antigen associated with MHC class I molecules in damaged keratinocytes. The exact nature of the antigen is unknown. This antigen could be a self-reactive peptide or an exogenous antigen, a certain protein, drug, infectious allergen etc. [65,66,67,68].

Initially, there is an increase in Langerhans epidermal cells, followed by the appearance of a superficial perivascular infiltrate consisting of lymphocytes and histiocytes. The chronic inflammatory condition causes damage to the epithelium, deposits of fibrinogen in the basement membrane and finally the destruction of the basal cell layer. The disease has a long evolution, with periods of remission and exacerbations [67,69].

Immunohistochemical studies also show an involvement of humoral immunity, with globular deposits of IgM, IgG and IgA in colloidal bodies. Increased serum titer of anti-desmoglein autoantibodies 1 and 3 also affects the integrity of adhesion glycoproteins. All these factors emphasize that OLP is the consequence of an immune reaction to a variety of systemic, topical or epithelial antigens and not a single disease [70,71].

Classic LP typically presents as pruritic, polygonal, violaceous flat-topped papules and plaques; many variants in morphology and location also exist, including oral, nail, linear, annular, atrophic, hypertrophic, inverse, eruptive, bullous, ulcerative, lichen planus pigmentosus, lichen planopilaris, vulvovaginal, actinic, lichen planus-lupus erythematosus overlap syndrome and lichen planus pemphigoides. Oral lesions of LP are classified by Scully and Lodi according to morphology into reticular, papular, plaque, atrophic, erosive and bullous [72,73].

The lesions of the oral mucosa are multiple, generally have a symmetrical distribution, especially on the jugal mucosa, in the area adjacent to the molars, and on the mucosa of the tongue, less often on the labial mucosa (lichenoid cheilitis) and gums (atrophic and erosive forms located on the gums manifest as DG). The clinical features of LP-associated DG vary according to the severity of the lesion (mild, moderate and severe). The mucosa acquires a bright red color, with small opaque gray plaques, which are found at the level of the attached gum [73,74]. The epithelium detaches with friction leaving a bright red underlying connective tissue which, on clinical examination, is very painful and bleeding. Associated symptoms include a burning sensation in the mouth and sensitivity to thermal changes. Highly spicy foods are not tolerated, and brushing is very difficult. For this reason, inspection of the lesion is difficult to perform, and patients often develop secondary, marginal gingivitis [75]. However, this clinical aspect of DG is not pathognomonic in erosive OLP.

In terms of pain sensitivity, patients with reticular lesions are often asymptomatic, but atrophic (erythematous) or erosive (ulcerative) OLP is often associated with a burning sensation and pain [75].

The most common type of OLP presents with keratotic lesions that have a reticular and whitish appearance. They are typically located on the lining of the buccal vestibule, and rarely on the palate and sublingual region. Gingival involvement is common, and in about 10% of cases OLP only manifests on the gingival mucosa. The typical presence is that of chronic DG delimited at the periphery by Wickham’s striae, a clinical aspect that facilitates the clinician’s diagnosis [74].

A higher malignant potential has been recognized for the atrophic and erosive forms of OLP. Lesions form on the back of the tongue and it is necessary to perform regular follow-up of patients up to an interval of three times a year. If dysplastic lesions are diagnosed following complementary examinations, the patient should be examined more frequently every 2–3 months. However, asymptomatic patients, mainly those with reticular form, may be evaluated annually. Signs that may indicate a malignant transformation, such as intensified symptoms and loss of homogeneity, should be carefully evaluated at each consultation. If the lesions alter their clinical appearance at follow-up visits, a biopsy should be considered immediately [74,76,77,78].

The positive diagnosis relies on the clinical aspect associated with histopathological findings.

For histopathological examination, a tissue biopsy is required, that must contain both the lesion and the perilesional area. Thus, one technique consists in taking two tissue samples: one of the perilesional tissue and the second that includes the area of the injured gum; both samples are collected through an elliptical tissue incision. The second method consists of taking lesional and perilesional tissue included in a single biopsy. In this situation, the tissue fragment will have to be dissected in the two areas: the lesion itself and the peripheral perilesional area [78].

Regardless of the method used for biopsy, perilesional tissue samples will be included in formalin and subjected to a routine histopathological examination. To prevent changes that may occur at the tissue or cellular level, tissue fragments taken from the lesion site itself are rapidly harvested on ice, double-fixed (osmium tetraoxide and glutaraldehyde) for electron microscopy examination or included in paraffin for processing for direct immunofluorescence technique [77].

It has been observed that indirect immunofluorescence and ELISA are generally unspecific in diagnosing desquamative gingivitis in lichen planus but they are much more useful in diagnosing autoimmune bullous disorders [78].

In the case of lichen planus, histopathological examination provides details of specific tissue changes, such as pseudoepitheliomatous hyperplasia, basal layer liquefaction, colloid bodies, shortened rete ridges, hyperkeratosis/ulceration and a band-like lymphocytic inflammatory infiltrate in the superficial chorion [36].

DIF may reveal features that are supportive for the diagnosis of OLP (linear deposition of fibrinogen at the basement membrane zone) [77].

Regarding the differential diagnosis, various lesions are clinically and histologically similar to oral lichen planus, and these are widely referred to as oral lichenoid lesions, as follows:

⁃ dental material-induced lichenoid lesions: various types of dental materials, such as amalgam, metals, composite and resin-based, are topographically associated with lichenoid reactions in the oral mucosa. In most cases, these contact allergies are due to a type IV/delayed hypersensitivity reaction. In these situations, a patch test using the suspected materials is useful for establishing the diagnosis [50,77,78,79,80,81,82,83].
⁃ drug-induced lichenoid damage: certain drugs, such as beta-blockers, non-steroidal anti-inflammatory drugs, antihypertensive agents (e.g., angiotensin converting enzyme inhibitors), dapsone, diuretics, oral hypoglycemic agents, gold salts and penicillamine have been reported to induce oral lichenoid damage. They often involve the lip and have a symmetrical distribution. An associated skin rash may suggest a drug-related injury [81,82,83,84].
⁃ erythema multiforme (EM): a reaction of hypersensitivity that occurs with damage to the skin or mucous membranes. Mucosal manifestations are common and have been reported in 25% to 70% of patients with EM. The oral mucosa is most commonly involved. The lesions begin as areas of erythema with edema that progress to erythematous plaques with bullous and erosive lesions, with pseudomembrane formation. Mucosal damage often occurs in combination with skin damage, which can provide a clue for diagnosis when typical and atypical targetoid lesions are found, often in an acral distribution. Isolated mucosal lesions may make diagnosis more difficult [81,82,83].
⁃ graft-versus-host disease: a multi-organ disease that occurs most frequently in patients who have undergone allogeneic stem cell transplantation. In the acute form, oral damage usually occurs in patients with more severe erosive skin disease. In chronic cases, oral lesions are usually similar to those of OLP, with white, reticulated, sometimes eroded plaques involving the oral mucosa, gums, and lips. Other mucosal membranes commonly affected include genital and eye mucosae [81,83,84].
⁃ cicatricial pemphigoid: a disease phenotype comprising several autoimmune bullous disorders characterized by subepithelial bullae and erosions with scarring of mucous membranes, skin or both and linear IgG deposition. The most commonly affected site of the oral mucosa is the gums, followed by the jugal mucosa and palate, and the most common presentation is DG. Oral damage usually presents as erythematous spots that progress to blisters, erosions and pseudomembranous lesions. Healing takes place slowly and often the visible scars in the oral mucosa are not obvious; white reticulated plaques similar to Wickham striae may be residual signs after healing of the erosions [66,81,82].
⁃ complex aphthosis/Behcet’s disease: it is a condition that occurs frequently on the non-keratinized mucosa. The disease often develops during adolescence or young adulthood and improves over time, with the appearance of reduced and milder outbreaks. Clinically, canker sores occur in the form of well-defined round or oval ulcers, often with a pseudomembranous base, on the oral and labial mucosa, dorsal and ventral surfaces of the tongue, soft palate and oropharynx [66,81,83].
⁃ pemphigus vulgaris: oral damage is usually the onset of pemphigus vulgaris and often precedes skin manifestations by several months. Irregular erosions and painful ulcerations are usually seen along the oral and labial mucosa, followed by the palate and tongue. These erosions develop on the mucosa with a healthy appearance, without erythema or surrounding edema. Intact blisters are rarely seen because they are fragile and prone to early rupture. The Nikolsky sign can be seen near an active lesion. Ulcers are persistent and do not heal spontaneously [66,82].
⁃ acquired bullous epidermolysis (EBA): damage to the mucosa may be manifested with erythema and erosions during the inflammatory phase and may progress over time to the loss of the lingual papillae and the healing of areas of the oral mucosa with the development of strictures. The distinguishing features of EBA are its refractory nature and poor response to treatment [82,83].

In terms of evolution, OLP is a chronic condition with undulating evolution that has relapses and remissions.

Treatment goals include reducing pain, healing ulcerative lesions, reducing the risk of cancer of the oral cavity, prolonging asymptomatic periods, and maintaining good oral hygiene and dental status [85].

**Figure 5:**
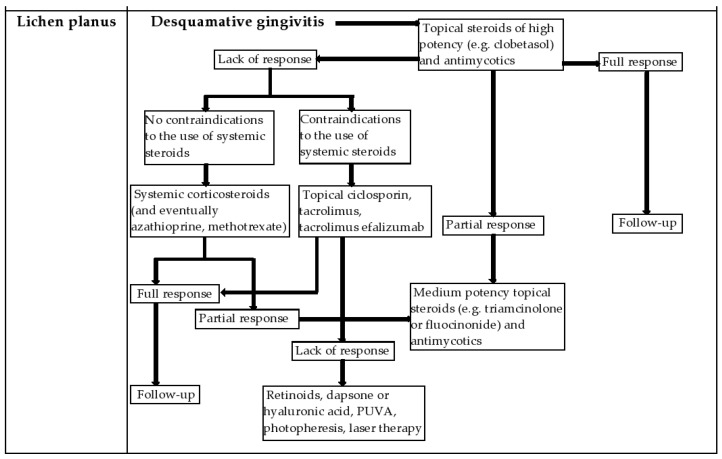
Treatment algorithm for desquamative gingivitis due to lichen planus.

In patients with OLP, establishing a bacterial plaque control program improved not only the frequency of gingival bleeding, but also the painful symptoms of the lesions and the general activity of the disease [50,85]. Figure 5 illustrates a treatment algorithm for the management of desquamative gingivitis due to lichen planus.

High-potency topical corticosteroids have been shown to be safe and effective for OLP lesions. In patients with atrophic-erosive form, a relatively equivalent efficacy of topical and systemic corticosteroid therapy was observed, demonstrating complete control of the disease in 68.2% of cases and 69.6%, respectively [50,86,87,88].

Local treatment with topical corticosteroids is initially recommended for symptomatic lesions, and in case of stationary or unfavorable evolution, systemic therapy may be considered. Limited lesions located on the fixed oral mucosa can be treated with ointments and creams [86].

In the case of severe and symptomatic forms of atrophic/erosive lichen planus, which do not respond to topical treatment and do not have contraindications for the administration of systemic steroids, intralesional and submucosal injection of corticosteroids should be considered [85,86].

In the presence of contraindications for systemic corticosteroids (breastfeeding, herpes infections, glaucoma, pregnancy, HIV, tuberculosis, diabetes mellitus or hypertension), other immunosuppressants and immunomodulatory agents are also recommended: calcineurin inhibitors such as cyclosporine, tacrolimus, tacrolimus efalizumab [84,85]. Other useful systemic agents are azathioprine and methotrexate [83].

In the absence of a therapeutic response, retinoids (tretinoin, isotretinoin, fenretinide), dapsone or hyaluronic acid may be used. Systemic retinoids are used in severe cases of LPO, but with significant side effects such as cheilitis sicca, increased serological levels of liver enzymes and triglycerides, teratogenicity. There are also several non-pharmacological therapeutic modalities such as PUVA therapy, photodynamic therapy or laser therapy [85,88].

Pain is often a significant symptom in erosive OLP. Topical anesthetics, such as viscous lidocaine, are frequently shown to be useful, especially in patients with low oral intake due to painful erosive lesions [87,88,89].

In the case of associated extra-oral lesions, it is important that the specialist in oral pathology work with the dentist, dermatologist and other specialists, as appropriate [85,90,91]. Annual monitoring is recommended and if relapses or new lesions are observed, additional biopsies may be performed and the patient will be re-evaluated more often [84,90].

Erosive LP lesions are often persistent and tend to respond poorly to therapy. The disease usually progresses over the years, and relapses are common even when maintenance therapy is given properly [87,89].

## 6. Management of the Patient with DG in the Dental Office and New Treatment Options

Dentists should provide patients with information about the disease and be responsible for helping them manage their symptoms through proper medication, proper oral hygiene, prevention of bacterial superinfection and proper nutrition [1,37]. Table 4 summarizes the management of desquamative gingivitis patient in the dental office.

Local treatment may be an option if the lesions are limited to the gums or oral mucosa. Topical agents that form a protective film at the level of erosions/ulcerations, as well as main preparations with anesthetic substances can be used to relieve pain. The general treatment of these systemic diseases, established following the specialized consultations in the dermatology and endocrinology clinics, will determine the healing of the lesions of the oral mucosa [1,37,90].

An alternative to topical agents is low-level laser therapy (LLLT) or also called photobiomodulation. Various types of laser devices for example diodes, helium–neon, ultraviolet, to name a few, can be used. These devices can be used with different parameters, such as output power, time of irradiation, dose and delivered to the oral lesion and repeated for several sessions [92]. They can be used due to their proposed anti-inflammatory effects, pain relief, and accelerated regeneration of damaged tissues, without demonstrating the adverse effects associated with drug intake treatment. This therapy has been advocated due to its anti-inflammatory, anti-bacterial and virucide effects but also for pain management and promoting a faster healing process in the affected areas, without the adverse effects that drugs have [93,94].

A recent meta-analysis that analyzed 17 studies has concluded that photobiomodulation determined an overall amelioration of clinical scores and significant pain reduction of treated areas, similar to topical corticosteroids, thus representing a viable alternative [95]. Although the preliminary results are promising, these alternative therapies should be further tested and compared to the classical therapy in order to assess their efficacy over time and for different disease stages.

Another important aspect that must be considered is the altered quality of life of patients with autoimmune diseases. Studies have highlighted the link between the immune system and the autonomic nervous system through the regulatory function that the latter has on the former through the parasympathetic and the sympathetic branches. If the autonomic nervous system is affected, it can generate an exacerbated pro-inflammatory response which can be usually encountered in autoimmune diseases [96]. Several studies have analyzed the impact of autoimmune diseases on quality of life and have concluded that these patients have an affected quality of life, increased stress, depression and anxiety [97,98,99,100,101]. Disease severity can further impact quality of life, as there is a positive correlation with this index [102]. Moreover, a recent meta-analysis reported increased salivary levels of cortisol, a biomarker for stress and anxiety, in oral lichen planus patients [103]. Taken together, this emphasizes the need for complex management of these patients, and more attention should be awarded to the psychological dimension of treatment.

## 7. Conclusions

DG is a pathological clinical manifestation of the gingival mucosa that is characterized by chronic desquamation of the gingival epithelium, erythema, ulceration and/or blistering. This is not a disease in itself, but rather the clinical phenotype of a group of diseases.

The most common causes are autoimmune bullous dermatoses, such as PV, BP, CP and LP with manifestations in the oral mucosa. Sometimes the lesions are strictly oral. For this reason, early diagnosis of autoimmune bullous disorders is imperative for clinicians. A thorough clinical examination, investigation of the patient’s history, complete blood count, biochemical tests as well as biopsies are necessary to formulate an appropriate early diagnosis and recommend early treatment with a beneficial response. Proper management of these patients can reduce side effects and lead to a better prognosis and quality of life for the patient.

## Figures and Tables

**Table 1:** Possible causes of desquamative gingivitis.

Causes of Desquamative Gingivitis	
Autoimmune diseases	Oral lichen planus ↑
	Cicatricial pemphigoid ↑
	Mucous membrane pemphigoid ↑
	Bullous pemphigoid
	Pemphigus vulgaris ↑
	Paraneoplastic pemphigus
	Epidermolysis bullosa acquisita ↓
	Lupus erythematosus
	Linear IgA disease
	Chronic ulcerative stomatitis
	Psoriasis ↓
Other systemic diseases	Erythema multiforme
	Graft-versus-host disease
	Toxic epidermal necrolysis ↓
	Pyostomatitis vegetans ↓
Irritant or allergic contact dermatitis- caused by	Mouthwash
	Toothpaste
	Dental materials
	Medication
Infections	Bacterial
	Viral
	Fungal
Hormonal imbalance	
Drug abuse	
Idiopathic	

↑ Most frequent; ↓ Least frequent.

**Table 2:** Target antigens specific for the most common autoimmune bullous dermatoses with oral involvement.

Autoimmune Bullous Dermatoses	Target Antigen
Pemphigus vulgaris	Desmoglein 1, desmoglein 3, desmocollin 3
Pemphigus foliaceus	Desmoglein 1
Paraneoplastic pemphigus	Desmoplakin I (250 kD), bullous pemphigoid antigen I (230 kD), desmoplakin II (210 kD), envoplakin (210 kD), periplakin (190 kD), plectin (500 kD), and a 170 kD protein
Cicatricial pemphigoid	Laminin V/VI, integrin, collagen, Alpha-1 (XVII) collagen chain
Bullous pemphigoid	Alpha-1 (XVII) collagen chain (formerly known as BP180/BPAG2) and dystonine (formerly known as BP230/BPAG1)

**Table 3:** Differential diagnosis of the main autoimmune bullous dermatoses with oral involvement.

Autoimmune Bullous Dermatoses	Differential Diagnosis
Pemphigus vulgaris	Bullous pemphigoid
	Erythema multiforme
	Linear IgA dermatosis
	Pemphigus erythematosus
	Pemphigus foliaceus
	Paraneoplastic pemphigus
	Drug-induced pemphigus
	Cicatricial pemphigoid
Bullous pemphigoid	Cicatricial pemphigoid
	Herpetiform dermatitis
	Drug-induced bullous dermatitis
	Erythema multiforme
	Linear IgA dermatosis
	Bullous epidermolysis
Cicatricial pemphigoid	Bullous pemphigoid
	Epidermolysis bullosa
	Linear IgA dermatosis
	Pemphigus vulgaris
	Erosive lichen planus

**Table 4:** Management of desquamative gingivitis patient in the dental office.

Management of the Patient with Desquamative Gingivitis in the Dental Office:	
Anamnesis. Careful clinical examination of the oral cavity by:	Assessment of the patient’s periodontal status: gingival retractions, exposure of dental roots, search of dental mobility, tooth loss, plaque control, use of plaque revealers. Assessment of dental status: detection of the presence and treatment of bone lesions. Evaluation of other mucosal lesions such as oral candidiasis, herpes gingivostomatitis etc.
2. Sanitizing the oral cavity by performing scaling and professional brushing	
3. Instructing the patient in order to achieve a rigorous oral hygiene (learning the correct brushing techniques—modified Bass technique—toothbrush placed at an angle of 45 degrees to the tooth surface, brushing twice a day, using electric or manual toothbrushes with soft bristles, use of mouthwash, oral rinses with alcohol-free antiseptic solutions).	
4. Patient follow up is mandatory because poor oral hygiene and treatment with immunosuppressive drugs can lead to increased bacterial colonization; it is necessary to periodically control the bacterial plaque, to determine the indices of gingival bleeding, to evaluate the pain and the evolution of the disease.	
5. It is recommended to establish a regular prophylactic protocol by using a local antiseptic (solution based on hydrogen peroxide applied twice a day); antifungal medication that will be administered at the time of diagnosis of the disease and later if a positive result is found on direct microscopic examination and culture of candida albicans.	
6. Establishing local or systemic curative treatment depending on the severity of the disease, by collaborating with doctors from the oral pathology and dermatology departments.	

## Data Availability

Not applicable.
